# A modified Whale Optimization Algorithm for exploitation capability and stability enhancement

**DOI:** 10.1016/j.heliyon.2022.e11027

**Published:** 2022-10-13

**Authors:** Kumeshan Reddy, Akshay K. Saha

**Affiliations:** Discipline of Electrical, Electronics & Computer Engineering, University of KwaZulu-Natal, 238 Mazisi Kunene Road, Durban, 4041, South Africa

**Keywords:** Algorithms, Convergence, Exploitation, Optimization, Whale optimization algorithm

## Abstract

Swarm-based Metaheuristic Optimization Techniques (MOT) are the dominant among all techniques, particularly owing to their simple nature and robust performance. The Whale Optimization Algorithm (WOA), a swarm-based MOT inspired by the hunting strategy of the humpback whale, has thus far shown promising results. However, like all MOT, the WOA is not without drawbacks. These demerits are a slow convergence rate and poor exploitation capability. This may prove to be problematic when applied to optimization problems requiring high precision results. Over the past few years, there has been proposed modifications to the conventional algorithm. However, experimental analysis highlights the need to further enhance the properties of the algorithm. This work proposes an enhanced WOA for exploitation capability and stability enhancement. The proposed algorithm introduces various modifications to the position update equations of the conventional algorithm, as well as a modified algorithm structure. The proposed algorithm was compared to various state-of-the-art MOT, as well as modified WOA proposed in recent literature. When applied to the CEC 2019 benchmark functions, the proposed algorithm produced the best result in 7 of the 10 test and had the most superior overall placement. When applied to practical problems, the algorithm once again demonstrated superiority. In addition, it was observed that the proposed algorithm exhibited a superior convergence rate to the other compared techniques.

## 1 Introduction

Metaheuristic Optimization Techniques (MOT), as the name suggests, are problem independent control techniques which has gained rapid popularity in the application of complex engineering problems. This can be attributed to their simplicity, flexibility, and capability to solve complex problems at a high efficiency rate. Metaheuristic techniques are based strongly on the concept of randomness, and search for optimal solutions based on diversification and intensification. Diversification is the scattered search of an entire search space and intensification is the search in a particular area of a search space [1]. Diversification and intensification are commonly referred to as exploration and exploitation respectively. MOT is based on various aspects of everyday life, such as the human body, the laws of physics and the behaviours of animals in their natural habitat [2]. Critical evaluation of the working processes of these aspects has allowed for accurate mathematical modelling of various nature-based occurrences. This in turn has been used to solve complex engineering problems successfully and optimally.

While there does not exist any definitive way to categorize MOT, it can usually be classified into four categories [2, 3]. These are swarm-based, physics-based, evolutionary based and human-inspired. From the four categories, swarm-based MOT is dominant among current literature. This can be attributed to their simple structure and effective results, even when attempting to optimize high dimensional problems. Delving into swarm-based MOT, is it observed that Particle Swarm Optimization (PSO) is the most established and utilized method for optimization. PSO is known for its merit of a fast convergence rate [4, 5] but suffers the demerit of being trapped at the local optima [4, 5, 6]. For a MOT to achieve strong results, there needs to exist a balance between exploration and exploitation. Several studies have been applied to the conventional algorithm to mitigate this demerit. One approach developed a new formula to dynamically change the inertial weighting factor [7]. While this did produce an improvement in results, there still existed room for improvement. The method outlined in [8] stochastically chooses a particle from the population, uses it to generate a new particle, and compared this particle to the worst particle in the swarm. If the new particle has a better fitness, it takes the place of the worst particle within the swarm. The approach in [9] developed a novel search equation to enhance the exploration capabilities of PSO. When tested on various benchmark functions, this method produced outstanding results.

Apart from PSO, another well utilized swarm-based MOT is the Artificial Bee Colony (ABC) algorithm. The ABC optimization algorithm is based on the foraging behaviour of a colony of bees. The ABC optimization technique is simple in structure and implementation and has thus far produced promising results. The merit of ABC is a strong exploration capability [10]. However, it suffers the demerit of a poor exploitation capability [11]. Due to the encouraging results presented in current literature, ABC has come under intense investigation, and subsequently various modifications have been developed. One such improvement proposed the modification of the initialization of the bees, which is conventionally done at random. This modification was based on the chaotic search. Further, inspired by Differential Evolution, the search equation of ABC was modified and written in terms of the position of the best bee, as well as the position of various other randomly chosen bee [11]. Two new search equations were presented in [12], which are also based on the position of the best bee, as well as the positions of various other bees chosen as random. The difference is that in this case, the previous position of the current bee is considered. A selective probability was used to choose between the two search equations, resulting in a superior balance between exploration and exploitation. A minor modification of these two equations is presented in [13], but this small change led to a much better result. In addition to the new equation proposed in [13], Orthogonal Learning was utilized to further enhance the results of the algorithm. While proving to be successful, this algorithm was highly complex in nature.

The Whale Optimization Algorithm (WOA) is a MOT which is inspired by the hunting tactic of the humpback whale [14]. Proposed by Mirjalili and Lewis in 2016, this relatively new MOT has shown promise in the optimization of complex engineering problems. This is largely due to the algorithm displaying the merit of a strong global search ability [15]. However, like all other MOT, the WOA suffers two critical demerits. These are a slow convergence rate and poor accuracy [16]. Since its inception, the WOA has come under investigation, albeit not extensively. In [17], Gaussian distribution strategies are used to simultaneously enhance the accuracy and convergence rate of the algorithm. However, this work only tests the proposed algorithm in 25 and 30 dimensions and utilizes the same search range across all test functions. The paper in [18] makes use of quadratic interpolation and a dynamic strategy to enhance the exploitation ability of the algorithm. This was aimed at improving the algorithm when attempting to solve large scale problems. The scholars in [19] proposed a modified technique for COVID-19 X-Ray image segmentation. The co-efficient vector A, as well as the constant value b are dynamically changed to improve both exploration and exploitation. When compared to various other modified versions of the conventional WOA, the proposed technique yielded a superior performance. In [20], a single dimensional swimming based WOA was proposed. This method was tested on a large range of dimensions, but the minimum dimension was 20. In [21], a Levy flight based mutation, along with a pattern search mechanism are integrated into the conventional WOA. The mutation enhances the exploration and exploitation capability of the algorithm, whereas the patter search improves convergence rate and stability. The proposed technique was utilized for parameter identification of solar cells and photovoltaic modules.

Considering the modification proposed in [22], a change to the algorithm structure is presented. This is along with a new position update equation for the encircling prey method. The new equation utilizes the positions of three mutually exclusive whale, which are different to the whale being updated. The algorithm structure change is in terms of a newly randomized number in the domain [0,1]. The positions of the whales are then updated according to the magnitude of this new randomized number. Hybridization of the WOA with other MOT have also been proposed in existing literature. In [23], the Grey Wolf Optimization algorithm, known to exhibit a strong local search ability, is integrated into the WOA (which lacks this aspect). However, the algorithm was tested using only one-dimension magnitude. The WOA was hybridized with a well-known evolutionary MOT, known as Genetic Algorithm, in [24].

In [25], the WOA was hybridized with the sine-cosine algorithm, a physics-inspired MOT. The proposed algorithm enhanced the exploration position update equation of the WOA via utilization of the position update equation of the sine-cosine algorithm, which makes use of four randomly generated numbers in the domain [0,1]. There exist two equations for the exploration search, with utilization determined by the magnitude of one of the random numbers in relation to a critical value. The authors in [25] applied the proposed algorithm to the IEEE 69-bus test system and compared to the conventional WOA. While producing an enhancement in the convergence rate and minor advancement in solution accuracy, the proposed algorithm was not rigorously tested on various functions and at various dimension magnitudes. The WOA was combined with simulated annealing, another physics-inspired MOT, in [26]. The proposed approach utilizes simulated annealing after the WOA has completed running. Further, the concept of mutation and tournament selection were added to the position update equations of the WOA. The algorithm in [27] introduces four operators into the conventional algorithms. These are differential evolution, density peak clustering strategy, nonlinear parameter design and opposition-based learning method. The proposed technique was tested on various benchmark functions, as well as the seismic inversion problem and compared to various modified WOA technique. The proposed algorithm generated superior results in terms of average value and standard deviation. Considering convergence, the proposed algorithm exhibits superiority after the completion of about 35 iterations. Quadratic interpolation and Levy flight is utilized in [28] to enhance the accuracy of microarray data classification. When compared to another modified WOA, the proposed algorithm generated a superior accuracy. However, no information concerning convergence rate was provided. In [29], the concept of correction factors are applied to the various position update equations of the conventional WOA. When tested on a range of benchmark functions and compared to other conventional algorithms, the proposed technique produced the overall best average result. The proposed algorithm was further applied to an adaptive fuzzy logic PID controller for load frequency control. The result proved to be remarkable, but was not fared against other algorithms, thereby comprising the validity of superiority.

As evident, there has been proposed modifications to the conventional WOA. However, it is observed that there still exists a lack of precision, as well as a sub-par convergence rate. Both exploitation and rate of convergence are critical parameters in the performance of optimization techniques. In numerous applications, even a change of a fraction of a percentage may yield large savings in resources. While the relevant proposed algorithm succeeded in their objectives, there still exists gaps in application. The authors in [18, 20] and [22] do not consider small dimension magnitudes, and only apply the proposed algorithm to classical benchmark functions (as opposed to modern functions). In [21], there was no comparison to any other MOT, even the conventional WOA. The article outlined in [23] improves slightly in this aspect, making comparisons with conventional algorithm. However, no proposed techniques were evaluated. Considering [19, 24] and [26], application was made only to one real-world engineering problem. Further, no application to CEC benchmarks functions, or any other functions, were made.

These deficiencies in current literature compromises the validation of the proposed novel ideas for global optimization, particularly in applications requiring high-precision results. An example of such is the gain values of PID controllers, where a low accuracy may result in sub-optimal controller performance. In electrical engineering, such problems are load flow analysis, economic load dispatch, and co-ordination of protection relays [30]. This correlates to optimal weight design of gear systems and machine scheduling in mechanical engineering [31]. In the civil and geotechnical engineering discipline, such examples are pile and rock design, and rock and soil mechanics [32]. There are also various optimization problems in other disciplines, like chemical engineering and computer engineering, where a high solution accuracy is imperative.

In this paper, various modifications are applied to the conventional position update equations of the WOA. Further, there is a change in the structure of the algorithm. Firstly, the stochasticity of the exploration search is improved. Then, via the use of parameters already contained within the algorithm, all three position update equations are modified. The proposed algorithm also allows the whales a chance to undergo a dual position update, if a criterion is met. Lastly, a critical fragment of the well-known ABC optimization algorithm is deployed in the proposed scheme. The aim of these updates is to mitigate the drawbacks of the conventional WOA, particularly in applications requiring high precision results. To demonstrate extensive testing, the proposed algorithm is applied to the CEC 2019 benchmark functions and compared to the conventional WOA, modified versions of such, as well as a recently proposed state-of-the-art technique. Further, to evaluate the true performance of the algorithm, the algorithm is applied to the optimal design of a pressure vessel. The rest of the paper is as follows. Section two describes the conventional WOA, including relevant equations and implementation procedure. Then, the proposed WOA is described in detail in section three. This provides a clear background into each modification proposed, as well as the resulting position update equations and algorithm structure. Section four presents the results of the experiments undertaken, as well as a critical analysis of such. Section fives concludes the research work done in the paper.

## 2 The Whale Optimization Algorithm

Whale optimization algorithm (WOA) is inspired by the hunting tactic of the humpback whale. The hunting strategy of the humpback whale is separated into three parts: searching, encircling and bubble-net attacking [14, 33, 34]. During searching, the humpback whales exchange information about prey to each other. This is to ensure that all the whales stay close to the prey. Consider the following [35, 36]:

(1) $$X_{i}\left(t\right)=\left[X_{i,1}\left(t\right),X_{i,2}\left(t\right)\ldots X_{i,D}\left(t\right)\right]$$

Where $X_{i}\left(t\right)$ is the current position of the $i^{th}$ whale and D is the number of search space dimensions. The position of the whales at the next sampling instant can be updated using three methods. The first method is via a random search. This is also known as exploration and is shown as [14, 35, 36]:

(2) $$X_{i}\left(t+1\right)=X_{r}\left(t\right)-A\left|C\times X_{r}\left(t\right)-X_{i}\left(t\right)\right|$$

Where $X_{r}\left(t\right)$ is the position of a whale chosen at random and A and C are coefficients. A is based on the current and maximum iteration numbers, as well as a random number in the range [0,1]. C is based only on a random number in the range [0,1]. It is important to note that the random numbers used in the evaluation of A and C are generated independently. The second method is to encircle the prey. To encircle the prey, each of the whales update their positions based on the best position found thus far. This update is represented as follows [14]:

(3) $$X_{i}\left(t+1\right)=X_{p}\left(t\right)-A\left|C\times X_{p}\left(t\right)-X_{i}\left(t\right)\right|$$

Where $X_{p}\left(t\right)$ is the best position found thus far (at iteration t). The third method is via the use of bubble net attacking. Bubble net attacking is a mathematical model used to imitate the spiral movement of the humpback whale [30, 31]. In bubble net attacking, the whales update their positions as follows [14, 35, 36]:

(4) $$X_{i}\left(t+1\right)=X_{p}\left(t\right)-A\left|C\times X_{p}\left(t\right)-X_{i}\left(t\right)\right|\;e^{bl}\times \cos\left(2\pi l\right)$$

Where b is a limited constant and l is a random number in the range [-1,1]. The method of position updating to be used is based on a random number P in the range [0,1], as well as the value of A. If P is less than 0.5 and the magnitude of A is greater than one, the whale positions are updated using encircling of the prey. If P is greater than 0.5 and the magnitude of A is greater than or equal to 1, the whale positions are updated randomly. Else, the bubble net attacking method of position updating is used [14].

**Figure 1:**
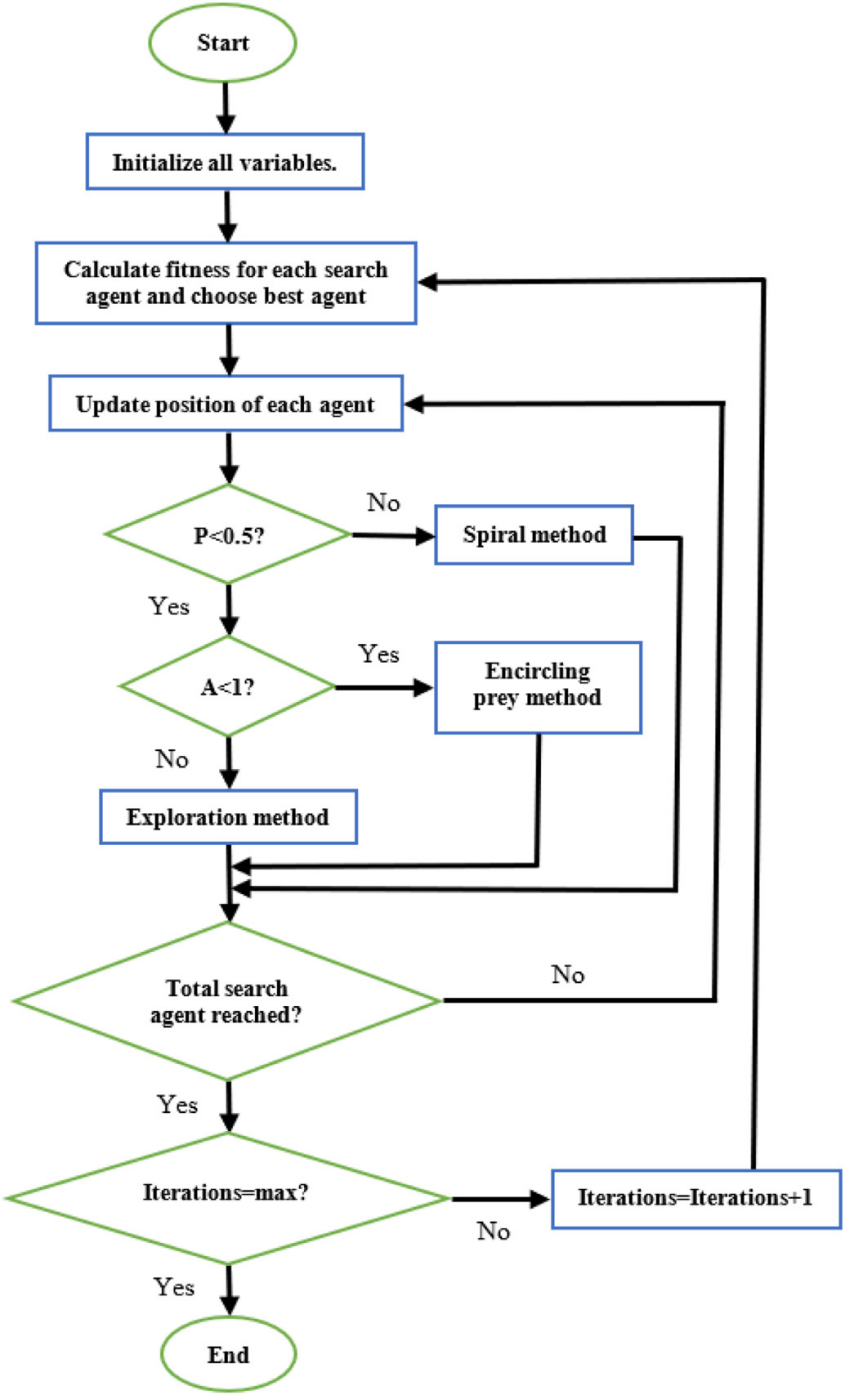
Structure of WOA [37].

Initially, the required parameters are defined. Then, each whale is given a random position. The fitness of each whale is calculated and the whale with the best fitness value is noted. The random numbers P and A are then generated. If the magnitude of A is less than 1 then the position of each whale is updated using (4). If P is less than 0.5 and the magnitude of A is greater than one, then the position of each whale is updated using (3). Lastly, if P is greater than or equal to 0.5, the position of each whale is updated using (2). After the update is completed, the fitness of each whale is calculated and replaces the current best fitness value (of that whale) if its value is superior to that of the current best. This continues until all iterations have been completed. Once this is so, the whale with the best fitness is said to be at the most optimal position [14]. The steps to execute the WOA can be seen in Figure 1 [37].

## 3 An enhanced Whale Optimization Algorithm

The proposed WOA introduces various modifications to the position update equations of the WOA, as well as a change to the general structure of the algorithm. The aim of such is the mitigate the two common demerits of the WOA: low accuracy and slow convergence, as well as to prevent possible local optima entrapment at higher dimension optimization problems.

To enhance the stochasticity of the exploration search method, the equation outlined in (2) is modified. This is via introduction of the position of another randomly chosen whale. This whale may be the same as the one already utilized in the current equation, or different. As in the original exploration search, the position of the current whale position is subtracted from the product of coefficient C and the second randomly chosen whale. The product of this value, as well as coefficient A, is added to the original equation.

The exploration search then becomes:

(5) $$X_{i}\left(t+1\right)=X_{r}\left(t\right)-A\left|C\times X_{r}\left(t\right)-X_{i}\left(t\right)\right|+A\left|C\times X_{r2}\left(t\right)-X_{i}\left(t\right)\right|$$

Where $X_{r2}\left(t\right)$ is the position of the second randomly chosen whale.

To further improve the search diversity and hence achieve an enhanced search accuracy, a fragment of the well-known ABC is utilized. In the conventional WOA, upon completion of all fitness values, each value is fared against the current best value. If a value is superior to the current value, it replaces the previous best value, and the corresponding whale positions now become the optimal positions. In the proposed technique, if the fitness value of a whale is not deemed superior to the current best, the position of that particular whale is updated as follows:

(6) $$X_{i}\left(t+1\right)=(X_{i}\left(t\right)+(\frac{t}{Max_{iter}})\times (X_{p}\left(t\right)-X_{j}\left(t\right))|$$

Where t is the current iteration number, $Max_{iter}$ is the maximum number of iterations and $X_{j}\left(t\right)$ is a randomly chosen whale from the population. The position of $X_{j}\left(t\right)$ must be different to $X_{i}\left(t\right)$.

**Figure 2:**
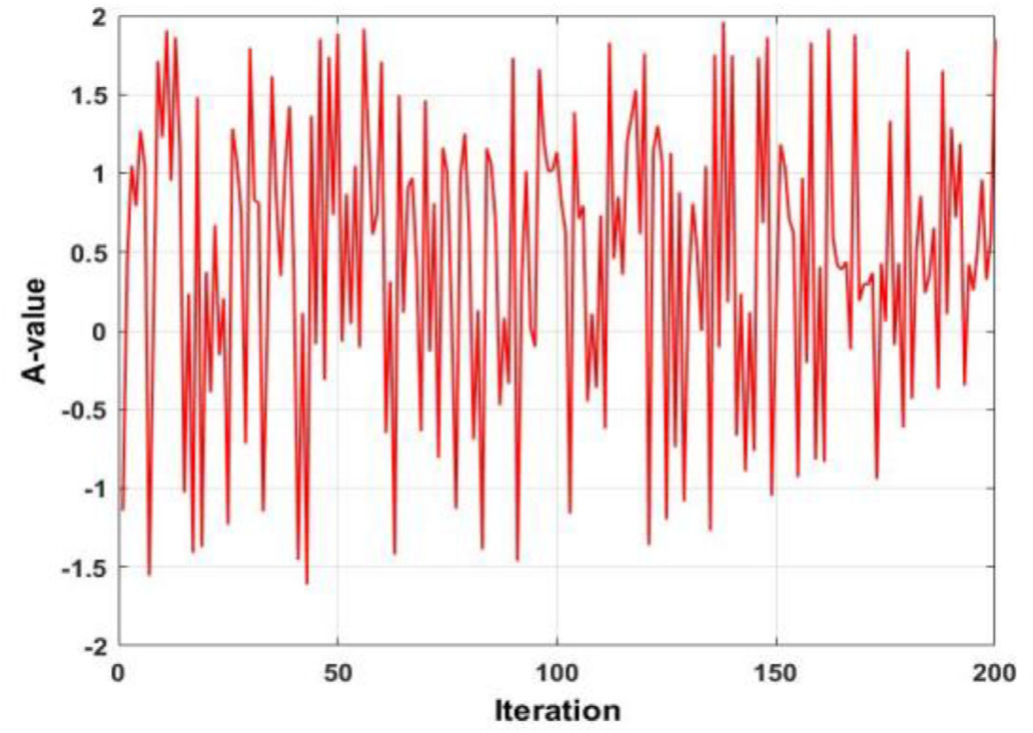
Behaviour of coefficient A.

**Figure 3:**
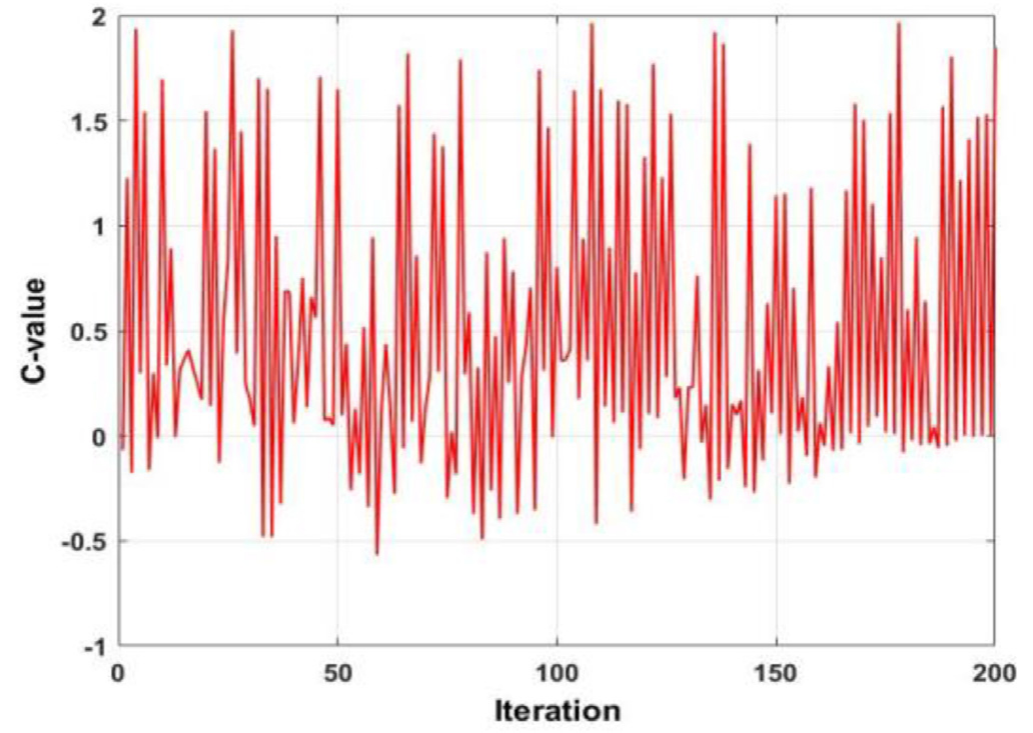
Behaviour of coefficient C.

It is observed that the coefficient A utilized in the conventional WOA is a function of a linearly decreasing number. This number is a function of the current iteration number, as well as the maximum number of iterations. However, another component of this coefficient is a stochastic number, which lies in the domain [0,1]. An identical phenomenon is observed with the coefficients C (utilized in the exploration and encircling prey search position update equations and l (utilized in the spiral search position update equation). The stochastic nature of A is shown in Figure 2. The magnitude of A can be seen to be oscillating between -1.5 and 2. The stochastic nature of coefficient C is shown in Figure 3. The response of C is nearly identical to A, with two differences. Firstly, the magnitude oscillates between -0.5 and 2 and secondly, the frequency of ripples seems to be more than that of the response of A. Considering the trends of coefficients A and C, it is observed that the effect of a linearly decreasing, or increasing number, on the effect of the WOA is yet to be investigated.

When applied to PSO, a linear increasing coefficient outperformed a linear decreasing one in terms of convergence rate and solution accuracy [38]. Therefore, a linearly increasing coefficient, which is a function of the current and maximum iteration numbers, is added to the second terms in (2) and (3), and the first term in (4). In [8], the concept of stochasticity was added to the cognitive and social constants, as well as the dynamic inertial weighting factor which are present in PSO. The results of this experiment produced a superior convergence rate to various other modified PSO algorithms. Utilizing this concept, a random number in the domain of [0,1] is added to the second terms in (2) and (3), and the first term in (4). Note that the three random numbers generated are unique to each other but may be equivalent in magnitude.

**Figure 4:**
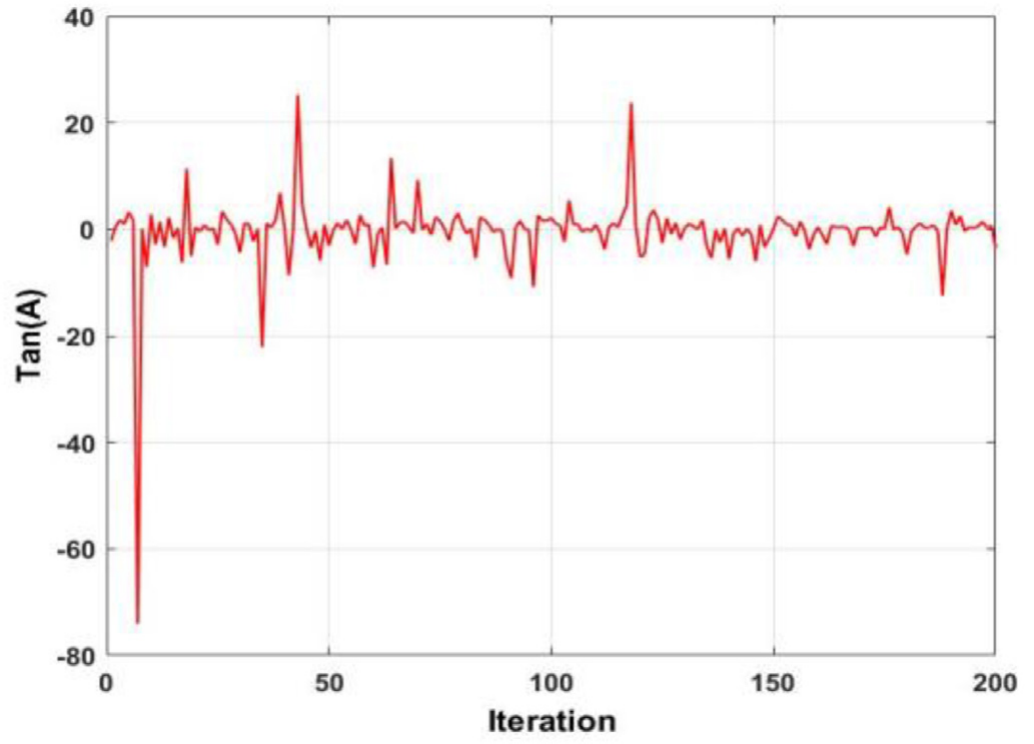
Behaviour of the tangent of coefficient A.

**Figure 5:**
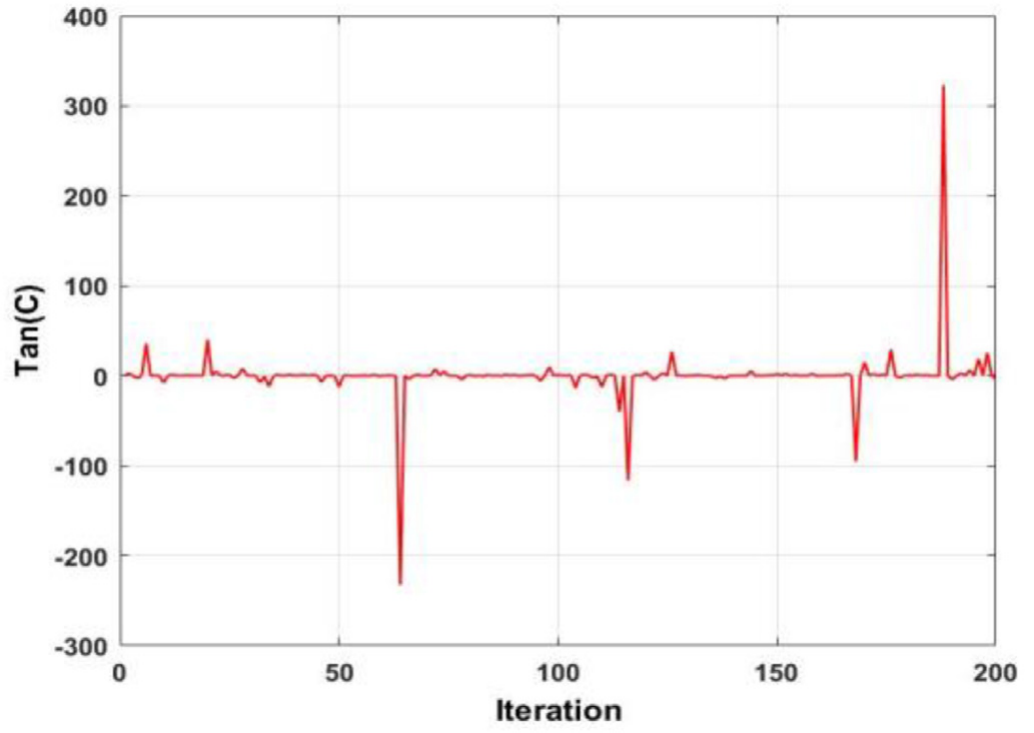
Behaviour of the coefficient of tangent C.

**Figure 6:**
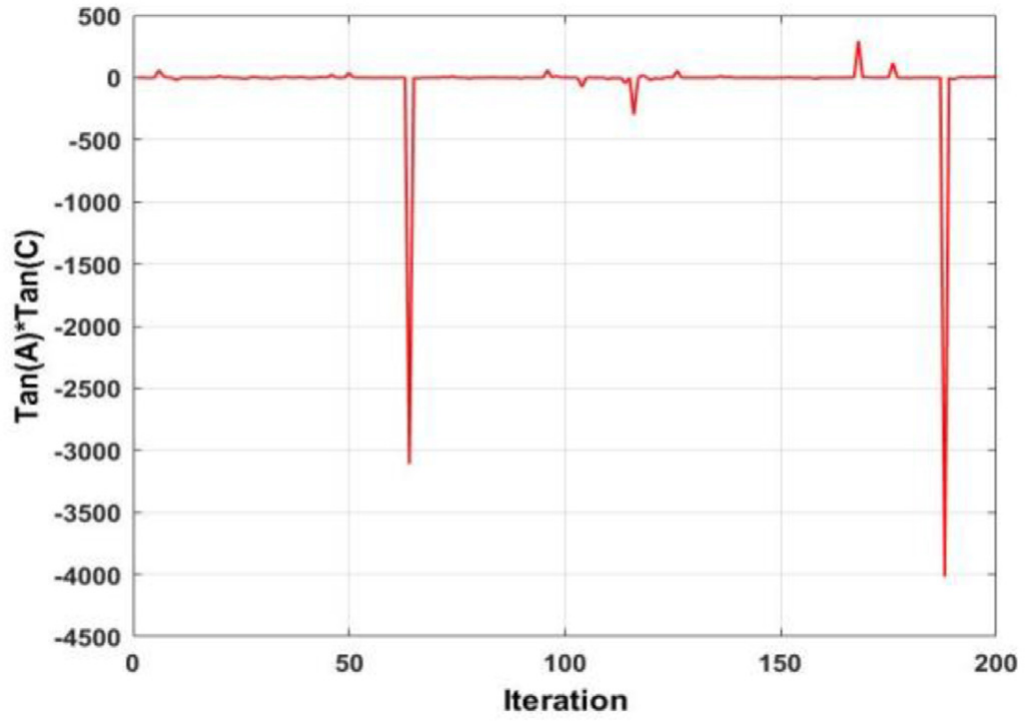
Behaviour of the product of the tangent of A and C.

To further enhance the exploitation capability of the WOA, the tangent of the two linearly decreasing coefficients A and C are utilized to create a new term in each equation. Figure 4 shows the plot of the tangent of coefficient A. It is observed that the value of the coefficient fluctuates around the zero point, both in the positive and negative. A similar phenomenon is seen in Figure 5, which displays the plot of the tangent of coefficient C. The difference, however, is that in Figure 5, there exists spikes of large magnitudes. Taking the product of both responses, Figure 6 is derived. As evident, Figure 6 is nearly identical to Figure 5, but displays exacerbated qualities.

Considering the exploration search position update equation, the product of the tangent of A and tangent of C, along with a randomly chosen whale will exacerbate the randomness of the search. Considering the encircling prey and spiral search position update equations, the product of the tangent of A and tangent C along with the position of the best whale will enhance the exploitation capability of the search equation. This will be achieved via the minor spikes, as observed in Figure 6. The larger spikes which are present will allow the algorithm to increase its capability of jumping out of the local optima, particularly at higher order optimization problems. Considering (2), the new term is the product of the position of the random whale, as well as the tangent of coefficients A and C. Considering (3) and (4), the new term is the product of the position of the best whale, as well as the tangent of coefficients A and C. The effect of dimension magnitude inclusion in position updating equations is not well researched. In several instances, it is observed that algorithms lose either their exploration or exploitation capability when attempting to optimize large scale problems. To mitigate this adverse effect, the inverse of the dimension magnitude is added to the second terms in (2) and (3), and the first term in (4).

Considering the various modifications that have been proposed, the new search equations are as follows:

Exploration method:

(7) $$\begin{array}{cc} X_{i}\left(t+1\right)={\;X}_{r}\left(t\right)-\left(1/dim\right)\times rand1\times \left(t/{\mathrm{Max}}_{iter}\right)\times A\left|C\times X_{r}\left(t\right)-X_{i}\left(t\right)\right| & +X_{r}\left(t\right)\times \tan\left(A\right)\times \tan\left(C\right) \end{array}$$

Encircling prey method:

(8) $$\begin{array}{cc} X_{i}\left(t+1\right)=X_{p}\left(t\right)-\left(1/dim\right)\times rand2\times \left(t/{\mathrm{Max}}_{iter}\right)\times A\left|C\times X_{p}\left(t\right)-X_{i}\left(t\right)\right| & +X_{p}\left(t\right)\times \tan\left(C\right) \end{array}$$

Spiral method:

(9) $$\begin{array}{cc} X_{i}\left(t+1\right)=\left(1/dim\right)\times rand3\times \left(t/{\mathrm{Max}}_{iter}\right)\times X_{p}\left(t\right)-\left|X_{p}\left(t\right)-X_{i}\left(t\right)\right| & e^{bl}\times \cos\left(2\pi l\right)+X_{p}\left(t\right)\times \tan\left(C\right) \end{array}$$

**Figure 7:**
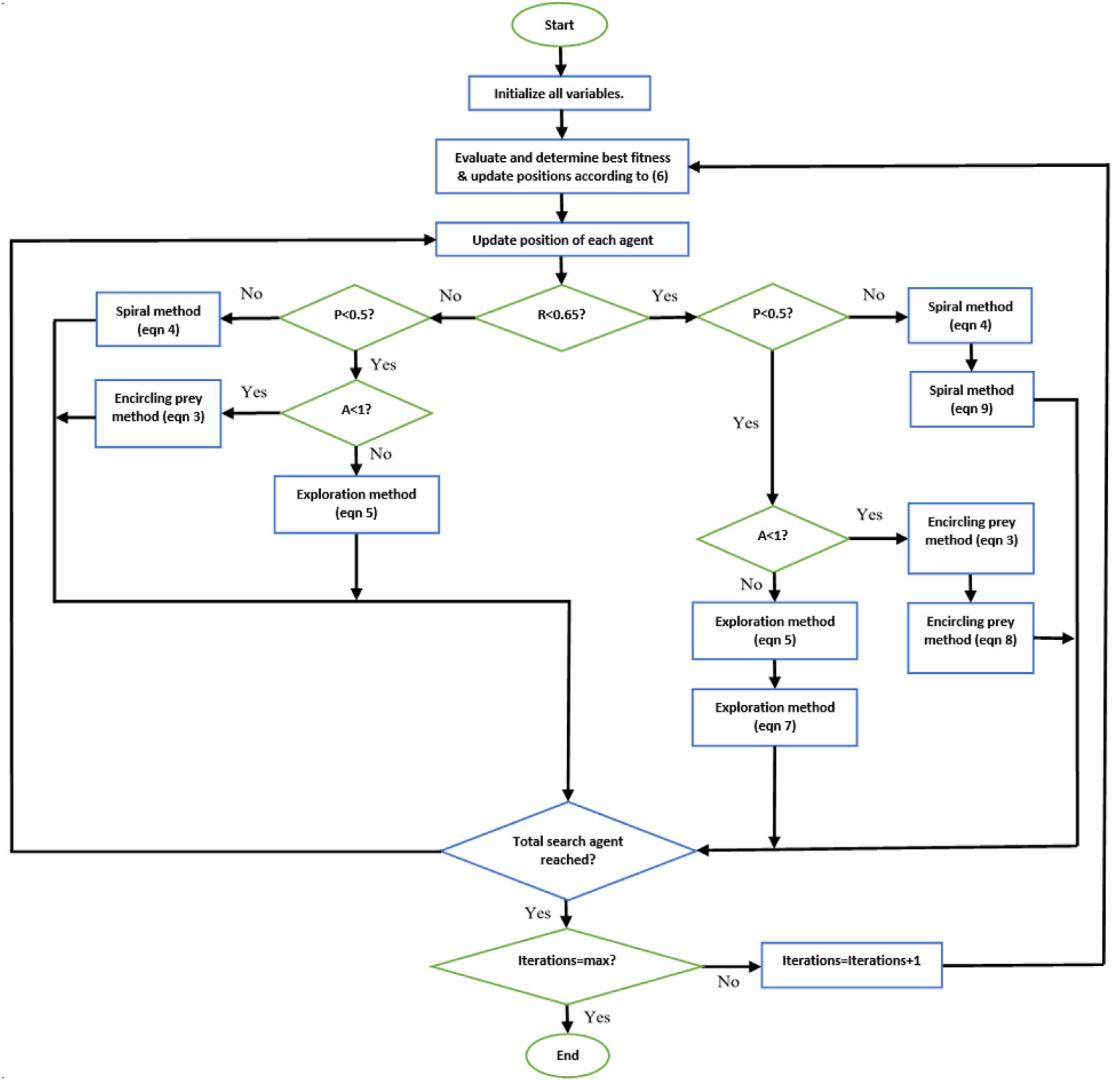
Structure of proposed WOA.

Where rand1, rand2, rand3 are random numbers in the domain [0,1], dim is the dimension magnitude of theoptimization problem, t is the current iteration number and $Max_{iter}$ is the maximum number of iterations. It is observed that there now exist two possible position search update equations for each of the exploration search, spiral search, and encircling prey. A random number in the domain [0,1] is defined. If this number is less than 0.65, then Eqs. (5), (3), and (4) are implemented. The choice of equations is determined based on the values of A and P, exactly like how is determined in the conventional WOA. Upon completion of this, (7), (8), and (9) are implemented. The choice of equations is determined based on the values of A and P, exactly like how is determined in the conventional WOA. Hence, in such scenario, execution of (7) will succeed (5), (8) will succeed (3), and (9) will succeed (4). If the randomly generated number is greater than 0.65, then only Eqs. (3), (4), and (5) are utilized. Once again, the choice of equations is determined based on the values of A and P exactly like how is determined in the conventional WOA. The structure of the proposed WOA, known as Enhanced Whale Optimization algorithm (EWOA) is therefore as shown in Figure 7. In Figure 7, R denotes a random number in the domain.

## 4 Experimental results and analysis

To validate the effectiveness of the proposed algorithm, the EWOA is applied to the well-known CEC2019 benchmark functions. A description of these ten functions can be found in Appendix A [39]. The EWOA is compared to the conventional WOA, as well as two modified versions of the conventional WOA. To ensure rigorous testing, the EWOA was also fared against the farmland Fertility Algorithm, a newly proposed MOT which has thus far delivered exceptional results.

In the first modified WOA, two new stochastic parameters are introduced. The parameters are as follows:

• $B=\frac{A}{\left(2\times rand\right)-1}$
• w=0.3+0.3×rand

In addition, the parameter C in the conventional algorithm is replaced with a value that is twice that of B. If a random number generated is less than that of B, the positions of the whales are updated by multiplying the current position by the addition of one and a random number in the domain [0,1]. Next, if the random number is less than or equal to 0.5, the encircling prey method is utilized. Finally, if the random number is less than 0.5, bubble net attacking is used. The parameter w is attached to the leader position in each equation.

The second modified WOA also introduced various new parameters, but these are not stochastic in nature. These are:

• $w=0.9-\left(0.9-0.7\right)\times {\left(\frac{It}{MaxIt}\right)}^{\left(\frac{1}{It}\right)}$
• $s=e^{\left(1-\frac{MaxIt}{MaxIt+1-It}\right)}$
• $v=0.6\times 2^{s}$
• $\delta =v\times \left(X_{best}-X_{t}\right)$

The three position update equations of the WOA then become:

• $X_{i}\left(t+1\right)=X_{r}\left(t\right)-wA\left|C\times X_{r}\left(t\right)-X_{i}\left(t\right)\right|$
• $X_{i}\left(t+1\right)=X_{p}\left(t\right)-A\left|C\times X_{p}\left(t\right)-X_{i}\left(t\right)\right|+\delta$
• $X_{i}\left(t+1\right)=X_{p}\left(t\right)-wA\left|C\times X_{p}\left(t\right)-X_{i}\left(t\right)\right|$$e^{bl}\times \cos\left(2\pi l\right)$

Owing to the stochastic nature of MOT, each algorithm was run 20 times. The results are given in terms of average value obtained, standard deviation, and convergence rate. The conventional WOA and MWOA1 were subject to the parameters presented in the original work [40]. This is also the case for MWOA2 [41] and the FFA [42]. The number of whales utilized in the EWOA is 1000, and the algorithm was subject to 100 iterations. Table 1 depicts the results obtained from application to the CEC2019 benchmark functions. From Table 1, it is observed that the EWOA produced the best average value in seven of the 10 benchmark functions. It is also evident that from all five algorithms, the proposed EWOA yielded the best overall rank. Considering only WOA, the proposed algorithm is superior in nine of the ten functions. Considering function 2, it observed that the EWOA exhibits a 0.28 % superiority over the next best algorithm (MWOA1). This corresponds to a large superiority of 27.43 % over the next best algorithm (WOA) for function 6. It is noted that FFA generates a fair number of good results but is still inferior to EWOA in terms of average ranking. Another advantage of the EWOA over the other algorithms is the concept of worst ranking. From Table 1, is it evident that the worst ranking attained by EWOA is third. This is superior to the FFA, WOA, and MWOA1 where the worst ranking attained is fourth, and superior to MWOA which attains a value of fifth. This points to an enhanced reliability of the proposed algorithm and verifies the EWOA as an effective algorithm for general optimization purposes.

**Table 1:** Performance analysis of proposed EWOA against other WOA for the CEC2019 benchmark functions.

Function		WOA	MWOA1	MWOA2	FFA	EWOA
1	Mean	1339570,194	51755,52062	**0**	4331170827	67948,78882
	Rank	4	2	**1**	5	3
	Std. dev	482949,83	5093,65	**0**	2484219709	12521,71
2	Mean	19,70131	17,41619	11929,59	17,47991	**17,36779**
	Rank	4	2	5	3	**1**
	Std. dev	0,47089	0,196649	4956,477	0,361856	**0,042862**
3	Mean	**12.7024**	**12.7024**	12.7065	**12.7024**	**12.7024**
	Rank	**2.5**	**2.5**	5	**2.5**	**2.5**
	Std. dev	**0**	**0**	0,000975	**0**	**0**
4	Mean	2767,298	1439,426	19627,67	**30,98509**	155,9416
	Rank	4	3	5	**1**	2
	Std. dev	1805,108	1358,2	5319,816	**14,81722**	64,82155
5	Mean	**8.1325**	**8.1325**	8.1518	**8.1325**	**8.1325**
	Rank	**2.5**	**2.5**	5	**2.5**	**2.5**
	Std. dev	**0**	**0**	0,008139	**0**	**0**
6	Mean	10,09868	10,73033	14,33454	10,25228	**7,92471**
	Rank	2	4	5	3	**1**
	Std. dev	1,16709	0,867645	1,079442	0,559376	**1,286647**
7	Mean	2097,158615	2097,15855	2199,621	**2097.1585**	**2097.1585**
	Rank	4	3	5	**1.5**	**1.5**
	Std. dev	0,000264127	0,000114708	67,75086	**0**	**0**
8	Mean	7,888065	7,89652	8,348015	**7,8796**	**7,8796**
	Rank	3	4	5	**1.5**	**1.5**
	Std. dev	0,026038	0,034719	0,261577	**0**	**0**
9	Mean	**4710,1035**	**4710,1035**	4711,26603	**4710,1035**	**4710,1035**
	Rank	**2.5**	**2.5**	5	**2.5**	**2.5**
	Std. dev	**0**	**0**	1,7626	**0**	**0**
10	Mean	20,90251	20,9092	21,11019	**20,9001**	20,90063
	Rank	**3**	4	5	**1**	2
	Std. dev	0,002697	0,028345	0,108713	**0**	0,001631
Average rank		31.5	29.5	36	23.5	**19.5**
Overall rank		4	3	5	2	**1**

The Wilcoxon Signed Rank Sum Test was carried out for the CEC 2019 benchmark functions. The test statistic was taken as the lower value of the positive and negative vales. For analysis purposes, a 5 % confidence interval was utilized. This corresponds to a critical value of 52. Table 2 shows the results of the Wilcoxon Signed Rank Sum Test for each of the benchmark functions. It is evident from the table that apart from function 2 against MWOA1 and FFA, the proposed algorithm displays significance in all possible scenarios. This validates the effectiveness of the EWOA to provide a statistically significant superiority to all of the compared algorithms.

**Table 2:** Wilcoxon signed rank sum test.

F	WOA	MWOA1	MWOA2	FFA
1	**0**	NA	NA	**0**
2	**0**	80	**0**	84
3	**0**	NA	**0**	NA
4	**0**	**0**	**0**	NA
5	NA	**2**	NA	NA
6	**0**	**0**	**0**	**2**
7	**0**	**0**	**0**	NA
8	**0**	**0**	**0**	NA
9	NA	NA	**0**	NA
10	**20**	**10**	**0**	NA

**Figure 8:**
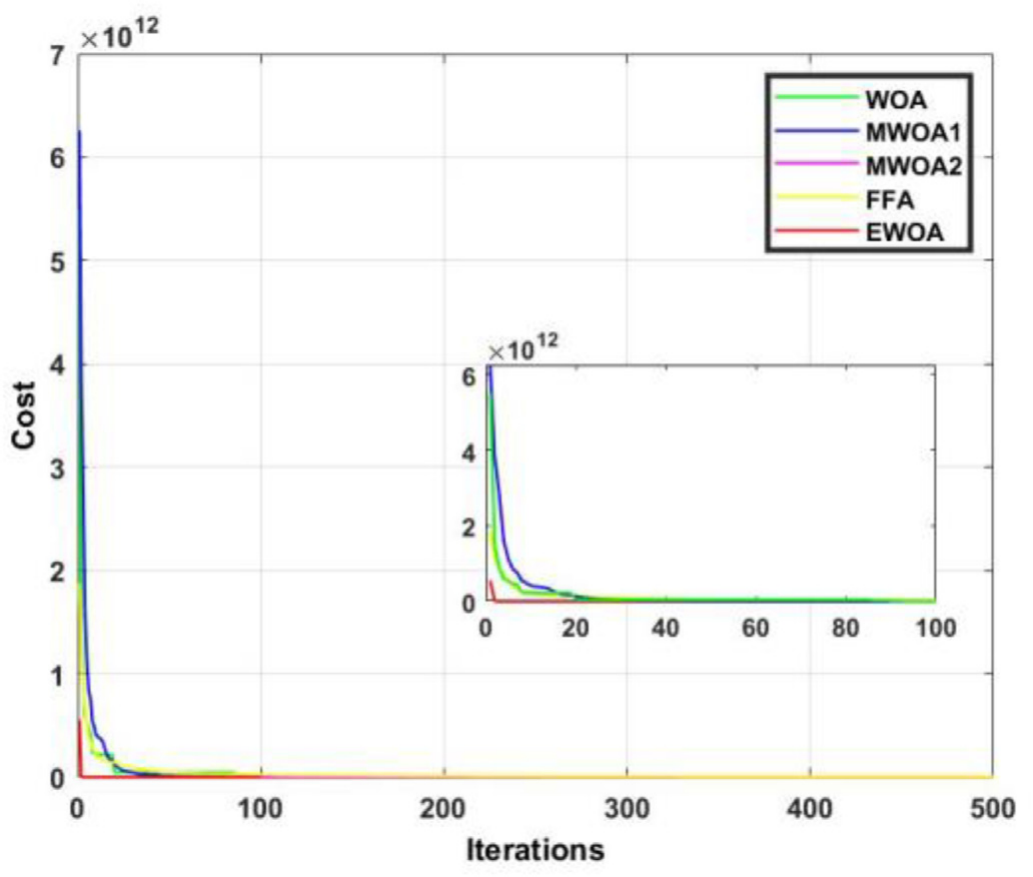
Convergence curves for F1.

**Figure 9:**
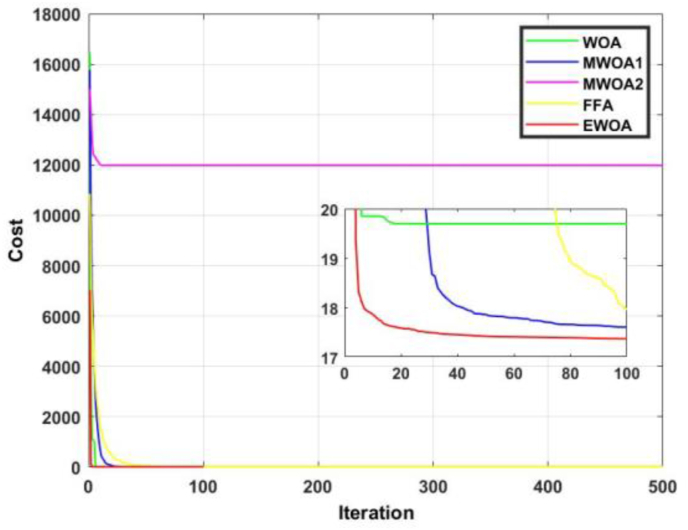
Convergence curves for F2.

The convergence curve for functions 1 and 2 are displayed in Figures 8 and 9. In Figure 8, it is observed that the proposed algorithm produced a superior result to FFA for the entire duration. In Figure 9, it is evident that the EWOA exhibits superiority over all the algorithms for the entire duration.

**Figure 10:**
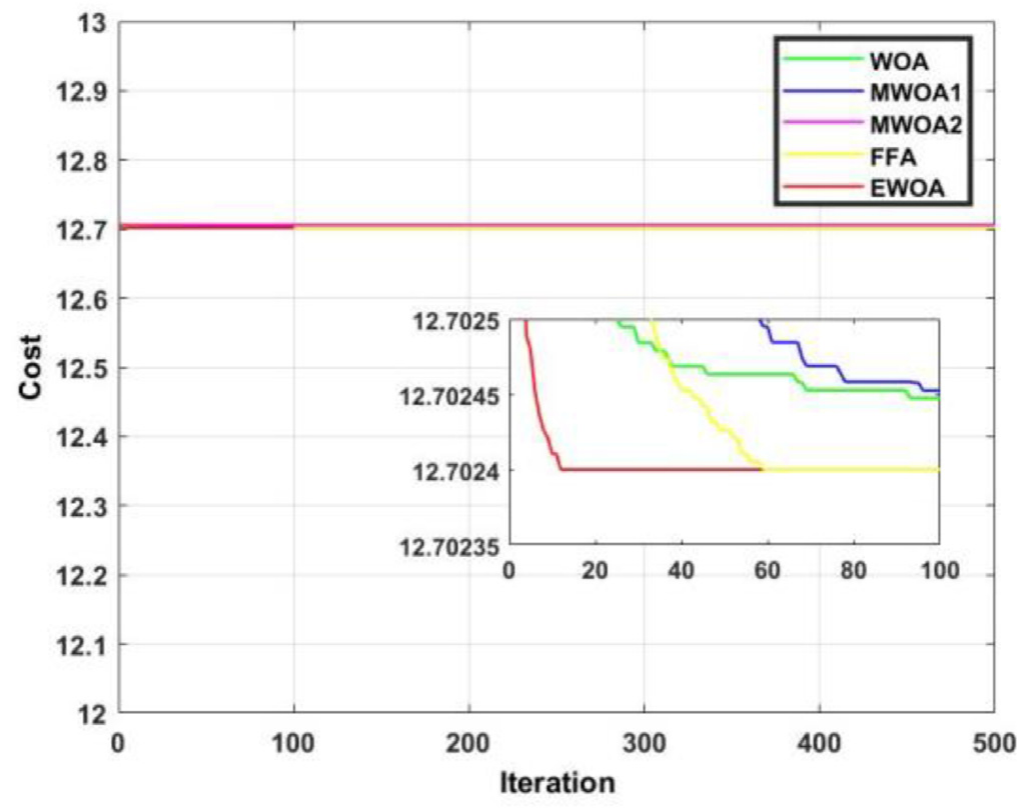
Convergence curves for F3.

**Figure 11:**
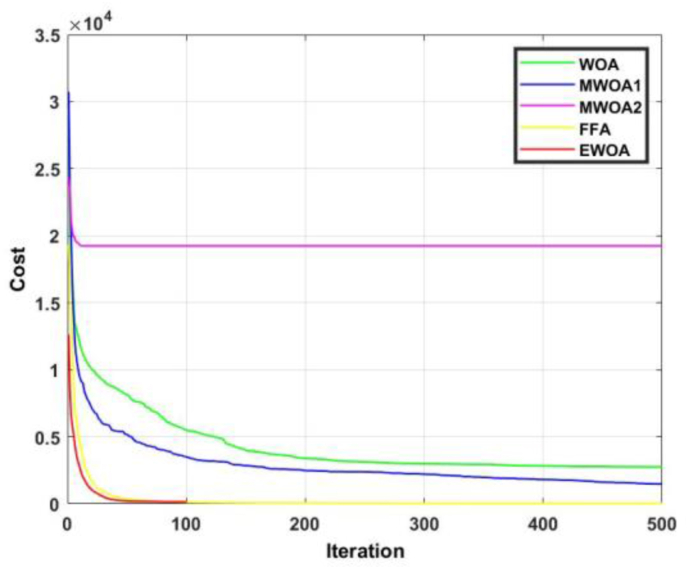
Convergence curves for F4.

**Figure 12:**
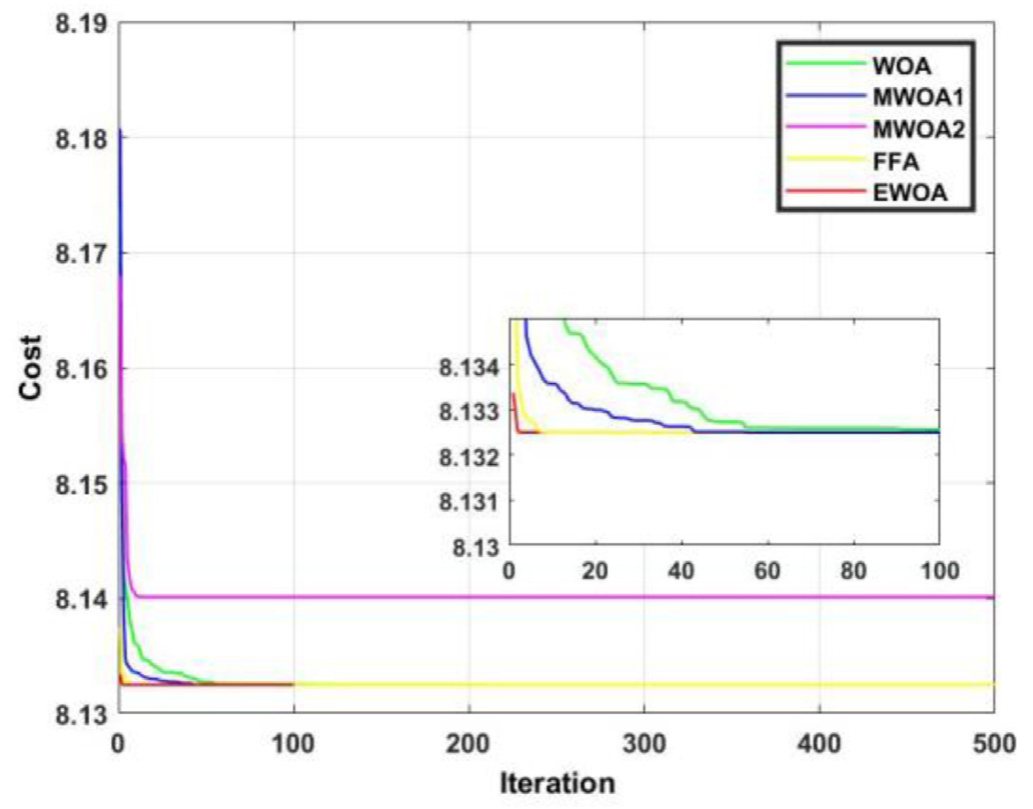
Convergence curves for F5.

The convergence curve for functions 3 and 4 are displayed in Figures 10 and 11. From Figure 11, despite most of the algorithms producing an identical result, the EWOA exhibited dominancy in that convergence occurred within less than 20 iterations. In Figure 12, the proposed algorithm was superior until about 60 iterations, after which the FFA reigned supreme.

**Figure 13:**
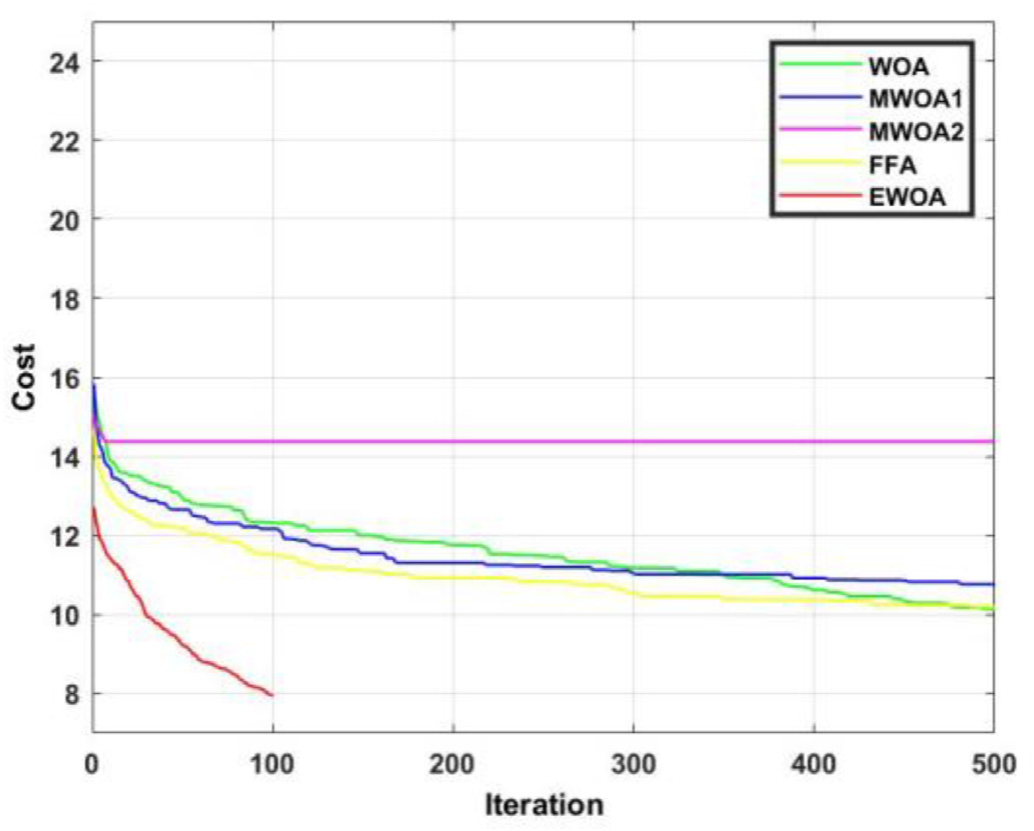
Convergence curves for F6.

The convergence curve for functions 5 and 6 are displayed in Figures 12 and 13. In Figure 12, the EWOA is superior to FFA for about 10 iterations, and to MWOA1 until 60 iterations have complete. In Figure 13, it is evident that the EWOA exhibits dominancy over all other algorithms for the entire duration.

**Figure 14:**
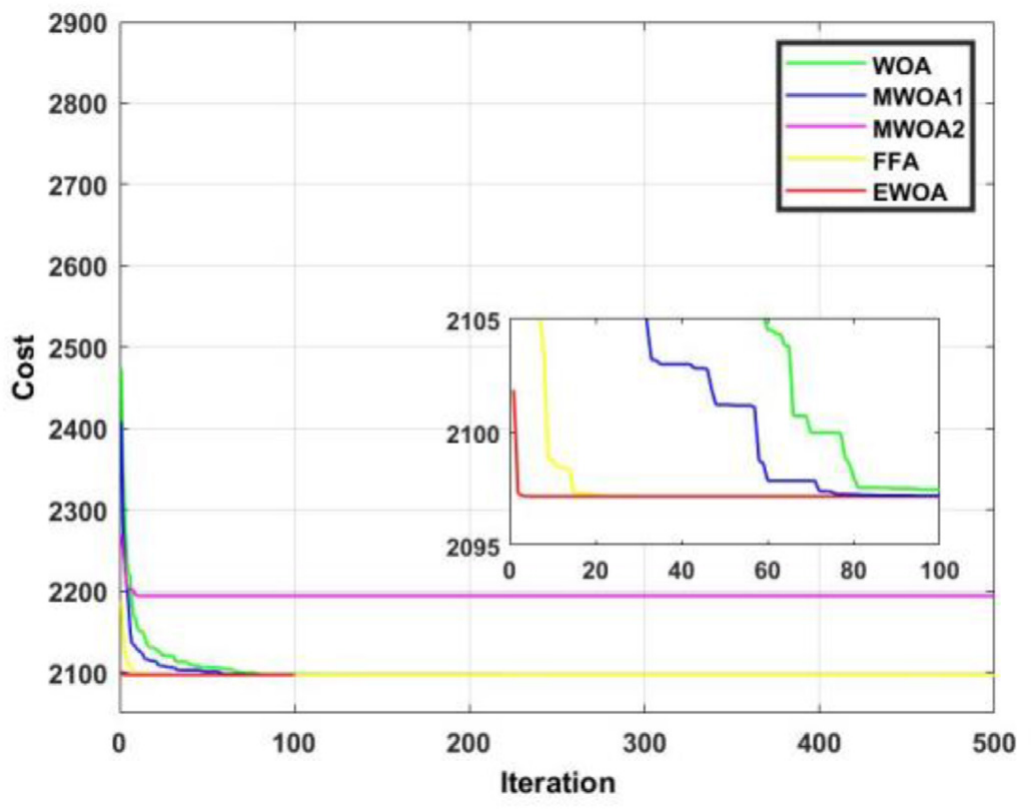
Convergence curves for F7.

**Figure 15:**
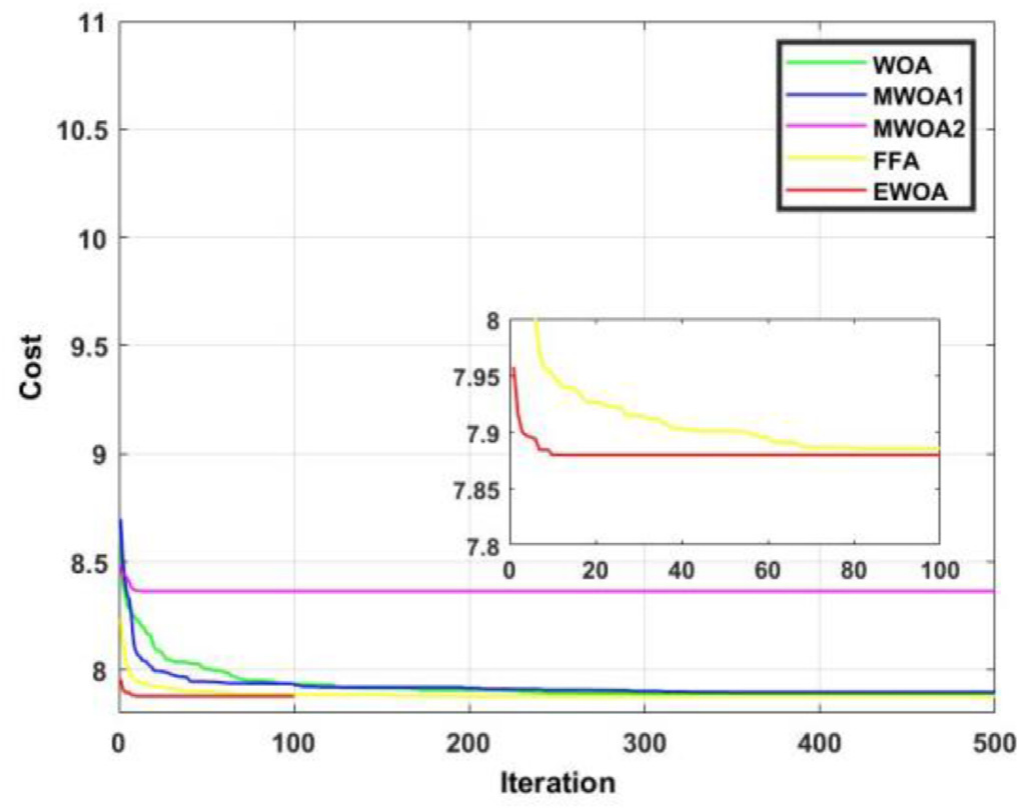
Convergence curves for F8.

The convergence curve for functions 7 and 8 are displayed in Figures 14 and 15. Figure 14 shows us that despite most of the algorithms producing an identical result, the EWOA converges much faster than any other algorithm. In Figure 15, it is evident that the proposed algorithm exhibited dominancy for the entire duration and converges after a mere 10 iterations.

**Figure 16:**
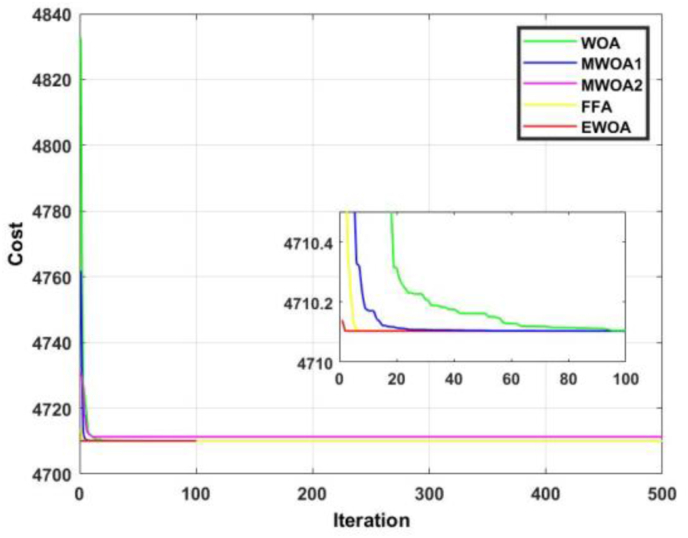
Convergence curves for F9.

**Figure 17:**
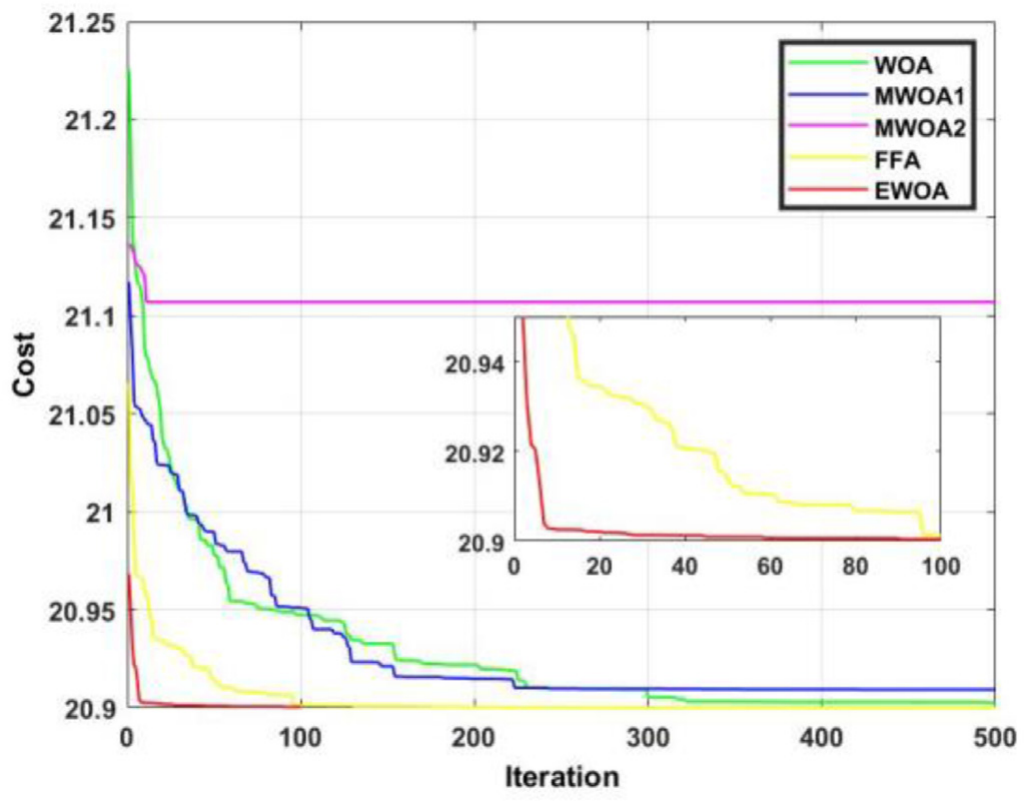
Convergence curves for F10.

The convergence curve for functions 9 and 10 are displayed in Figures 16 and 17. In Figure 16, it can be observed that the proposed algorithm produced the best convergence rate. In Figure 17, despite the EWOA faring second to FFA, the proposed algorithm exhibited superiority until 100 iterations.

**Figure 18:**
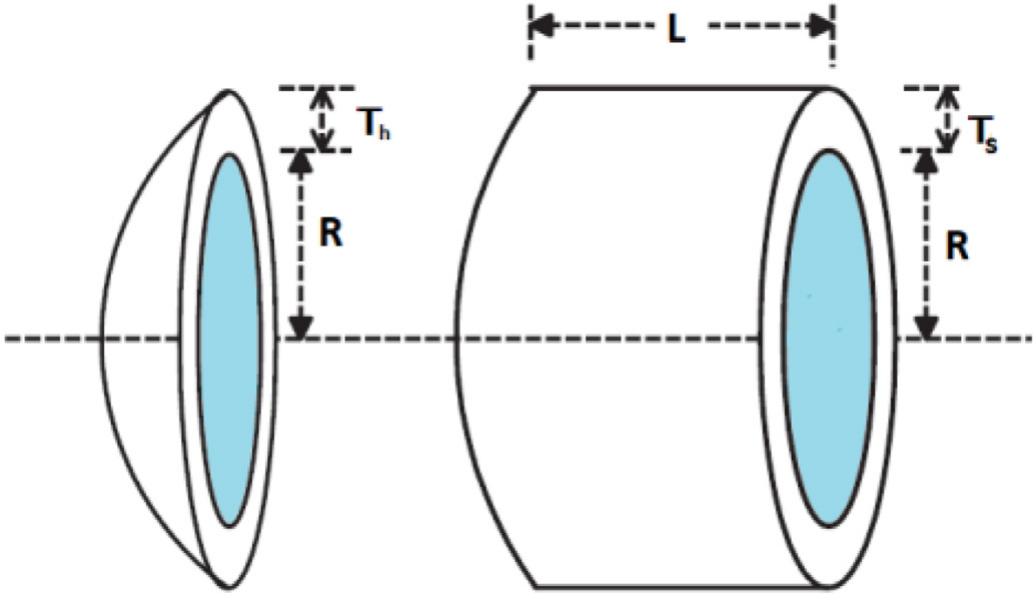
Structure of pressure vessel [44].

An important aspect of algorithm efficacy validation is application to practical engineering optimization problems. The proposed EWOA is applied to the design of a pressure vessel, a well-known constrained optimization problem used to determine the efficacy of optimization techniques. The structural makeup of a pressure vessel can be observed in Figure 18, where T1 is the head thickness, T2 is the thickness of the shell, L is the length of the cylindrical section of the vessel and R denotes the inner radius [43]. The ideal optimization cost of the pressure vessel design is zero.

The cost function of the pressure vessel is expressed as [45]:

$$f\left(x\right)=0.6224x_{1}x_{3}x_{4}+1.7781x_{2}x_{3}^{2}+3.1661x_{1}^{2}x_{4}+19.84x_{1}^{2}x_{3}$$

The design is subject to the following constraints [45]:

• $-x_{1}+0.0193x_{3}\leq 0$
• $-x_{2}+0.00954x_{3}\leq 0$
• $-\pi x_{3}^{2}x_{4}-\frac{4}{3}\pi x_{3}^{2}+1296000\leq 0$
• $x_{4}-240\leq 0$

Where $x_{1}$, $x_{2},x_{3},x_{4}$ denote $T_{s}$, $T_{h}$, R and L respectively. The design is subject to the following constraints. The range of the design variables are [45]:

• $1\leq x_{1}x_{2}\leq 99$
• $10\leq x_{3}x_{4}\leq 200$

Upon application of the said problem to the conventional WOA, MWOA and EWOA, the results obtained are as seen in Table 3.

**Table 3:** Performance analysis for design of pressure vessel.

Algorithm	Average	Std. Dev.
FFA	37397.72	0
MWOA1	9047.74	465.14
EWOA	**8810.96**	**17.34**

As evident from Table 3, despite producing a standard deviation of zero, the FFA struggles to escape from the local optima, thereby producing a significantly poorer solution to MWOA1 and EWOA. The EWOA produced the best average value, this being 2.62 % superior to MWOA. This correlates to a standard deviation superiority of greater than 2500 %

## 5 Conclusion and future works

This research work proposed an enhanced Whale Optimization Algorithm for optimization of complex engineering problems. The aim of such enhancement was to improve the search accuracy of the algorithm, as well as the stability of such. This is imperative for applications whereby precision results are valued, such as optimization of PI controllers, where incorrect controller tuning may result in unacceptable suboptimal performance. The proposed algorithm introduced various components to the position update equations of the WOA, as well as a change to the structure of the algorithm. Further, an aspect of the Artificial Bee Colony optimization algorithm was incorporated into the WOA. The proposed algorithm was applied to the CEC2019 benchmark functions and compared to the conventional WOA, modified versions of such, and the new Farmland Fertility Algorithm. The results show that the proposed algorithm produced the best result in 7 of the 10 functions. Further, the EWOA generated the best overall ranking. In addition, the reliability of the proposed method can be validated via observation that the poorest ranking of the proposed algorithm was third, lower than any other compared algorithm. When applied to the optimal design of a pressure vessel, the proposed algorithm yielded significantly superior results to both the MWO1 and FFA. However, investigations revealed that the EWOA required a significant number of whales in order to prove superior. Further, based on the structure of the algorithm outlined in Figure 7, there could exist instances whereby the whales will undergo a dual position changes within one iteration. This may put a strain on the RAM of the PC being used, and as a result may not be able to successfully be executed on PC's with poor random-access memory. Further, these two aspects contribute the time taken to execute the algorithm, which is higher than that of the other compared algorithms. However, this is somewhat compensated for by the requirement and subsequent use of a significant lower number of iterations.

Owing to the superior search accuracy of the proposed algorithm, it can be hypothesized that the EWOA will outperform the conventional WOA, as well as other MOT, in the tuning of PI controllers. This forms the basis for the future scope of work. The doubly fed induction generator is the most utilized generator in wind energy conversion systems. However, owing to direct grid connections, this generator struggles to meet the stringent levels of grid code requirements. Since wind energy conversion systems are required to remain connected to the grid during the case of voltage dips and unbalances, optimal design of PI controller for the generator is critical. Therefore, the design of PI controller for control of the doubly fed induction generator under the influence of voltage unbalances will be studied in the future. Another important application to be considered in the future is the optimal placements of overcurrent relays in a distribution system. With the increase in demand for electrical energy, and subsequent expansion of distributions systems, optimal placements of such devices are crucial for safe and efficient electrical supply.

## Declarations

### Author contribution statement

Kumeshan Reddy: Conceived and designed the experiments; Performed the experiments; Analyzed and interpreted the data; Contributed reagents, materials, analysis tools or data; Wrote the paper.

Akshay K. Saha: Analyzed and interpreted the data; Contributed reagents, materials, analysis tools or data; Wrote the paper.

### Funding statement

This research did not receive any specific grant from funding agencies in the public, commercial, or not-for-profit sectors.

### Data availability statement

No data was used for the research described in the article.

### Declaration of interest's statement

The authors declare no conflict of interest.

### Additional information

No additional information is available for this paper.

## Figures and Tables

**Table:** 

Function number	Function name	Dimension	Range	Optimum
F1	STORN’S CHEBYSHEV POLYNOMIAL FITTING PROBLEM	9	[-8192,8192]	1
F2	INVERSE HILBERT MATRIX PROBLEM	16	[-16384,16384]	1
F3	LENNARD-JONES MINIMUM ENERGY CLUSTER	18	[-4,4]	1
F4	RASTRIGIN’S FUNCTION	10	[-100,100]	1
F5	GRIENWANK’S FUNCTION	10	[-100,100]	1
F6	WEIERSRASS FUNCTION	10	[-100,100]	1
F7	MODIFIED SCHWEFEL’S FUNCTION	10	[-100,100]	1
F8	EXPANDED SCHAFFER’S F6 FUNCTION	10	[-100,100]	1
F9	HAPPY CAT FUNCTION	10	[-100,100]	1
F10	ACKLEY FUNCTION	10	[-100,100]	1
